# Deriving spatially explicit direct and indirect interaction networks from animal movement data

**DOI:** 10.1002/ece3.9774

**Published:** 2023-03-26

**Authors:** Anni Yang, Mark Q. Wilber, Kezia R. Manlove, Ryan S. Miller, Raoul Boughton, James Beasley, Joseph Northrup, Kurt C. VerCauteren, George Wittemyer, Kim Pepin

**Affiliations:** ^1^ Department of Geography and Environmental Sustainability University of Oklahoma Oklahoma Norman USA; ^2^ Department of Fish, Wildlife and Conservation Biology Colorado State University Colorado Fort Collins USA; ^3^ United States Department of Agriculture, Animal and Plant Health Inspection Service, Wildlife Services National Wildlife Research Center Colorado Fort Collins USA; ^4^ Forestry, Wildlife, and Fisheries, Institute of Agriculture University of Tennessee Tennessee Knoxville USA; ^5^ Department of Wildland Resources and Ecology Center Utah State University Utah Logan USA; ^6^ Center for Epidemiology and Animal Health United States Department of Agriculture, Animal and Plant Health Inspection Service, Veterinary Service Colorado Fort Collins USA; ^7^ Archbold Biological Station Buck Island Ranch Florida Lake Placid USA; ^8^ Savannah River Ecology Laboratory Warnell School of Forestry and Natural Resources University of Georgia South Carolina Aiken USA; ^9^ Wildlife Research and Monitoring Section Ontario Ministry of Natural Resources and Forestry Ontario Peterborough Canada

**Keywords:** African swine fever, chronic wasting disease, continuous‐time movement models, Global Positioning System (GPS), interaction, social network

## Abstract

Quantifying spatiotemporally explicit interactions within animal populations facilitates the understanding of social structure and its relationship with ecological processes. Data from animal tracking technologies (Global Positioning Systems [“GPS”]) can circumvent longstanding challenges in the estimation of spatiotemporally explicit interactions, but the discrete nature and coarse temporal resolution of data mean that ephemeral interactions that occur between consecutive GPS locations go undetected. Here, we developed a method to quantify individual and spatial patterns of interaction using continuous‐time movement models (CTMMs) fit to GPS tracking data. We first applied CTMMs to infer the full movement trajectories at an arbitrarily fine temporal scale before estimating interactions, thus allowing inference of interactions occurring between observed GPS locations. Our framework then infers indirect interactions—individuals occurring at the same location, but at different times—while allowing the identification of indirect interactions to vary with ecological context based on CTMM outputs. We assessed the performance of our new method using simulations and illustrated its implementation by deriving disease‐relevant interaction networks for two behaviorally differentiated species, wild pigs (*Sus scrofa*) that can host African Swine Fever and mule deer (*Odocoileus hemionus*) that can host chronic wasting disease. Simulations showed that interactions derived from observed GPS data can be substantially underestimated when temporal resolution of movement data exceeds 30‐min intervals. Empirical application suggested that underestimation occurred in both interaction rates and their spatial distributions. CTMM‐Interaction method, which can introduce uncertainties, recovered majority of true interactions. Our method leverages advances in movement ecology to quantify fine‐scale spatiotemporal interactions between individuals from lower temporal resolution GPS data. It can be leveraged to infer dynamic social networks, transmission potential in disease systems, consumer–resource interactions, information sharing, and beyond. The method also sets the stage for future predictive models linking observed spatiotemporal interaction patterns to environmental drivers.

## Graphical abstract

This study developed a method to estimate absolute animal interactions in space and time using continuous‐time movement models (CTMM) fit to GPS tracking data, which set up the foundation for understanding various ecological processes

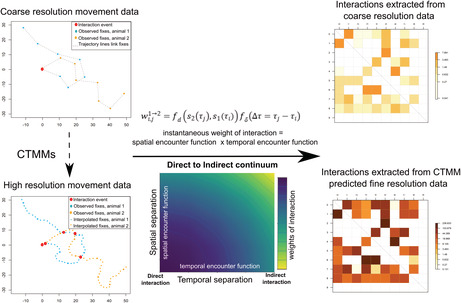

## 1 INTRODUCTION

Quantifying spatiotemporal animal interactions is fundamental for understanding drivers of social organization, infectious disease transmission, and predator–prey relationships (Albery et al., 2021; Raybeck, 2014; Webber & Vander Wal, 2019). Both direct (same place, same time) and indirect (same place, different time) interactions (Robitaille et al., 2018) are important in these contexts. In predator–prey systems, understanding direct physical contact as well as indirect interactions (how predators follow the trajectory of preys) has important implications for determining optimal foraging behavior, predicting predator–prey dynamics (Krivan, 1997), and estimating the influence of the “landscape of fear” on prey behavior (Coleman & Hill, 2014). In social species with hierarchical organizations, direct and indirect interactions among individuals can capture the fundamental components of social organization, the structure of the leadership hierarchy, and the underpinnings of collective behavior (Herbert‐Read et al., 2013). In infectious disease systems, spatiotemporal patterns of direct or indirect interaction drive pathogen transmission dynamics, prevalence, and ultimate epidemic size (Albery et al., 2021). However, gathering empirical data on spatiotemporally explicit animal interactions is often challenging in the field. Several technologies, such as camera traps, ultra‐high‐frequency proximity loggers, and Global Positioning System (“GPS”) telemetry, can be used to estimate direct and indirect interactions. GPS telemetry provides rich spatiotemporally explicit information on animal movement and is widely used to inform animal conservation and management (Kays et al., 2015), particularly for identifying individual–environment interactions (Fieberg et al., 2010). The ubiquity of GPS data, especially among widely studied, large‐bodied terrestrial wildlife, also provides the opportunity to capture unprecedented information on the spatiotemporal context surrounding animal interactions.

Despite the emerging tracking technology, such as solar‐powered GPS tags and Advanced Tracking and Localization of Animals in real‐life Systems (ATLAS) that allow the collection of high‐resolution movement data, many studies still use GPS collars with relatively low resolution (e.g., every 30 min or 1 h). Positional fix rate limitations often lead to the underestimation of interaction rates, as shown in disease transmission estimation from GPS collar data (Yang, Boughton, et al., 2021). Explicitly understanding ecological processes like epidemiologically relevant interactions or short‐lived predator–prey interactions requires interaction estimates at a fine temporal scale. Advancing our ability to estimate temporally continuous and spatially explicit interaction patterns at a landscape scale remains a key objective for improving ecological inferences about interaction‐based processes and developing methods to extract such information from GPS telemetry data. To address this limitation, several methods have been used to estimate interaction for disease systems from GPS data. For example, one approach uses home range overlap as a proxy for interaction rates (Kenward et al., 1998), but this strategy can miss fine‐scale spatiotemporal interaction patterns and is strongly dependent on methodology (Robert et al., 2012). Another approach uses co‐location of animals within predefined spatial and temporal buffers (Long et al., 2022; Robitaille et al., 2018), but this strategy can introduce errors when measuring interactions only at fixed time points or mis‐specified thresholds (Yang, Boughton, et al., 2021).

Continuous‐time movement models (CTMM; Calabrese et al., 2016; Johnson et al., 2008) offer a bridge to overcome the limitations in estimating animal interactions introduced by the discrete nature of GPS data (Manlove et al., 2022). CTMMs use telemetry observations to describe a continuous‐time movement process which in turn allows the models to naturally accommodate different temporal scales, predict potential unobserved movements during the tracking period, and provide a basis for simulating movement trajectories. Thus, these models provide a unique opportunity to improve the estimation of absolute animal interaction rates in continuous space and time (Dougherty et al., 2018). Recent advances have mathematically formalized ecological encounters in continuous time (Gurarie & Ovaskainen, 2013), inferred interaction location distributions from CTMMs (Noonan et al., 2021), and inferred direct and indirect interactions from continuous movement data in disease systems (Richardson & Gorochowski, 2015; Wilber et al., 2022). However, extending these approaches to (1) formally and flexibly account for direct and indirect interactions across ecological contexts and (2) apply directly to commonly collected movement data remains important knowledge gaps. Further, CTMM‐based approaches and traditional methods, for example, empirical summary of interactions using only observed fixes, have yet to be compared directly.

**FIGURE 1:**
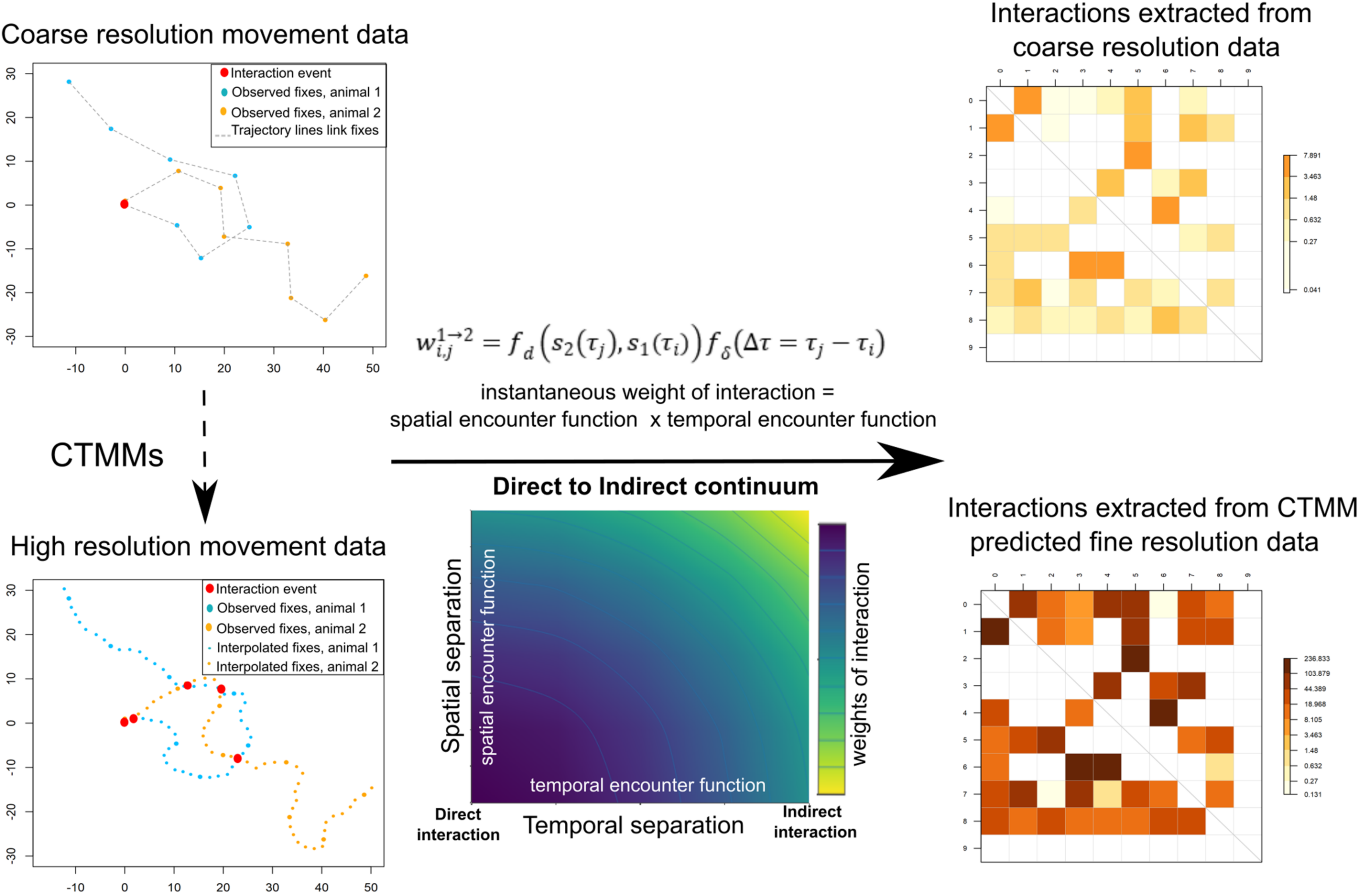
Schematics of CTMM‐interaction method.

Here, we develop a method for applying CTMMs to quantify the weights of each interaction event across a continuum of direct to indirect interactions based on a spatial and temporal encounter function using GPS data (i.e., CTMM‐Interaction; See schematics in Figure 1). We use simulated movement data to evaluate the CTMM‐Interaction method for quantifying spatiotemporally explicit direct and indirect interactions. We then assessed the performance of the method using the simulated movement data downsampled at different temporal resolutions. To demonstrate how the method can be employed in different animal movement systems and parameterized relative to different interaction periods of interest (i.e., in relation to different pathogen life‐histories), we applied it to two contrasting host–pathogen systems, African swine fever (ASF) in wild pigs (*Sus scrofa*) and chronic wasting disease (CWD) in mule deer (*Odocoileus hemionus*) in the western USA. Our CTMM‐interaction method allowing for comparable estimates of absolute interaction rates across systems with different propensities for indirect transmission can be modified to account for biological details affecting interactions and identify interactions that are missed when only the observed fix rates are used for interaction estimation.

## 2 MATERIALS AND METHODS

### 2.1 Constructing CTMM‐Interaction networks

The first step in our approach is to choose a CTMM that is appropriate for the data and movement behavior of the study species and fit that CTMM to the observed GPS data. The parameterized CTMM is then used to predict movement trajectories at a fine resolution in between GPS fixes for each collared individual. Fitting system‐specific CTMMs has received extensive coverage in the literature (Calabrese et al., 2016; Hooten & Johnson 2017; Johnson et al., 2008, etc.), and it is beyond our current scope. Instead, our emphasis is on the biological inference we can make about interaction processes given a parameterized CTMM.

Let *s*_*k*_(*t*) denote the continuous‐time movement trajectory for the *k* ^*th*^ individual as generated by the CTMM, where *s* is a two‐dimensional vector of the animal's spatial coordinates, and *t* indicates a focal position in time. Instantaneous direct and indirect interactions occur when two individuals are co‐located within a certain distance at the same time (direct interaction) or at different times (indirect interaction) based on the continuous movement outputs. From these identified co‐locations, our method builds direct or indirect spatial proximity networks.

We assume that the spatial distance between animal *1* at time $\tau_{i}$ and animal *2* at time $\tau_{j}$ could be calculated as the distance *d*_*1,2*_ between locations *s*_*1*_($\tau_{i}$) and *s*_*2*_($\tau_{j}$). The calculation of $w_{i,j}^{1\to 2}$, defined as the instantaneous weight of interaction experienced by animal *2* at time $\tau_{j}$ from animal *1* at time $\tau_{i}$, depends on the distance *d*_*1,2*_ and *∆τ*, the elapsed time separating $\tau_{j}$ and $\tau_{i}$. It is given by

$$w_{i,j}^{1\to 2}=f_{d}\left(s_{2}\left(\tau_{j}\right),s_{1}\left(\tau_{i}\right)\right)f_{\delta }\left(∆\tau =\tau_{j}-\tau_{i}\right)$$

where $f_{d}\left(.\right)$ is a spatial encounter function (Gurarie & Ovaskainen, 2013) that determines the spatial gap defining an interaction, and $f_{\delta }\left(.\right)$ is a temporal encounter function that determines the temporal gap of the interaction. An interaction kernel $K^{1\to 2}$ experienced by animal 2 from animal 1 consists of the instantaneous weight of interaction at all timesteps:

$$K^{1\to 2}=\left[\begin{array}{ccc} w_{1,2}^{1\to 2} & \cdots & w_{1,n}^{1\to 2}\\ \vdots & \ddots & \vdots \\ 0 & \cdots & w_{n,n}^{1\to 2} \end{array}\right]$$

See the toy example of the computation of interaction kernels in Section 1: Appendix S1.

### 2.2 Accounting for different ecological interactions

The key advance of our approach is the recognition that framing interactions in terms of an interaction kernel $K^{1\to 2}$ composed of a spatial and temporal encounter function allows us to simultaneously account for direct and indirect interactions and generalizes previous theory on ecological encounters (Noonan et al., 2021; Robitaille et al., 2018). For example, let $f_{\delta }\left(\Delta \tau \right)$ be a Dirac delta function $\delta \left(\Delta \tau \right)$ (essentially, an indicator function, here taking on the value 1 at ∆τ=0 to represent direct interaction and 0 at all other temporal lags) for a generic consumer–resource interaction and $f_{d}\left(s_{2}\left(\tau_{j}\right),s_{1}\left(\tau_{i}\right)\right)=\gamma \phi \left(s_{2}\left(\tau_{j}\right),s_{1}\left(\tau_{i}\right)\right)$ where γ is the encounter rate with units area per time and $\phi \left(s_{2}\left(\tau_{j}\right),s_{1}\left(\tau_{i}\right)\right)$ is an encounter kernel with units per area. A reasonable choice for $\phi \left(s_{2}\left(\tau_{j}\right),s_{1}\left(\tau_{i}\right)\right)$ might be a uniform circular distribution in space (e.g., Gurarie & Ovaskainen, 2013) that accounts for locational errors of the GPS device or the area of awareness for the two interacting animals (e.g., the distance at which a consumer can detect a resource). Then, $w_{i,j}^{1\to 2}=\gamma \phi \left(s_{2}\left(\tau_{j}\right),s_{1}\left(\tau_{i}\right)\right)\delta \left(\Delta \tau \right)$ and $h_{\mathrm{ji}}\left(\tau_{j}\right)=\int \gamma \phi \left(s_{2}\left(\tau_{j}\right),s_{1}\left(u\right)\right)\delta \left(\tau_{j}-u\right)\mathrm{du}=\gamma \phi \left(s_{2}\left(\tau_{j}\right),s_{1}\left(\tau_{j}\right)\right)$ yields a direct rate of interaction between consumer *i* and resource *j* at time $\tau_{j}$. This approach generalizes the formalization of encounter rates as described in Gurarie and Ovaskainen (2013) by illustrating that direct interaction rate is a special case where the temporal encounter function is a Dirac delta function. Alternatively, if we were interested in the rate of interaction of individual *j* with scent markings left by individual *i*, we could let the spatial encounter function $f_{d}\left(s_{2}\left(\tau_{j}\right),s_{1}\left(\tau_{i}\right)\right)=\beta \phi \left(s_{2}\left(\tau_{j}\right),s_{1}\left(\tau_{i}\right)\right)$ where β is an acquisition rate with units area per time and $f_{\delta }\left(\tau_{j}-\tau_{i}\right)=\lambda \left(\tau_{i}\right)S\left(\tau_{j}-\tau_{i}\right)$ where $\lambda \left(\tau_{i}\right)$ is a scent deposition rate at time *i* and $S\left(\tau_{j}-\tau_{i}\right)$ is a scent survival function (i.e., a monotonically decreasing function between 1 and 0). Then, $h_{\mathrm{ji}}\left(\tau_{j}\right)=\int \beta \phi \left(s_{2}\left(\tau_{j}\right),s_{1}\left(u\right)\right)\lambda \left(u\right)S\left(\tau_{j}-u\right)\mathrm{du}$ is the total interaction rate of individual *j* with individual *i*'s past and current scent markings, accounting for a continuum of direct and indirect interaction. This formalizes and generalizes approaches like those developed by Richardson and Gorochowski (2015) and Wilber et al. (2022). Section 2: Appendix S1 illustrates examples of weighted interactions in other systems. Parameterization of the interaction kernel depends on the combination of the spatial definition of interactions in the study system (i.e., direct or proximate interaction) and error in locational measurements. Code for manipulating the analyses is available at https://github.com/Anni‐Yang/ctmm‐interaction.

### 2.3 Assessment of CTMM‐Interaction method using simulations

**FIGURE 2:**
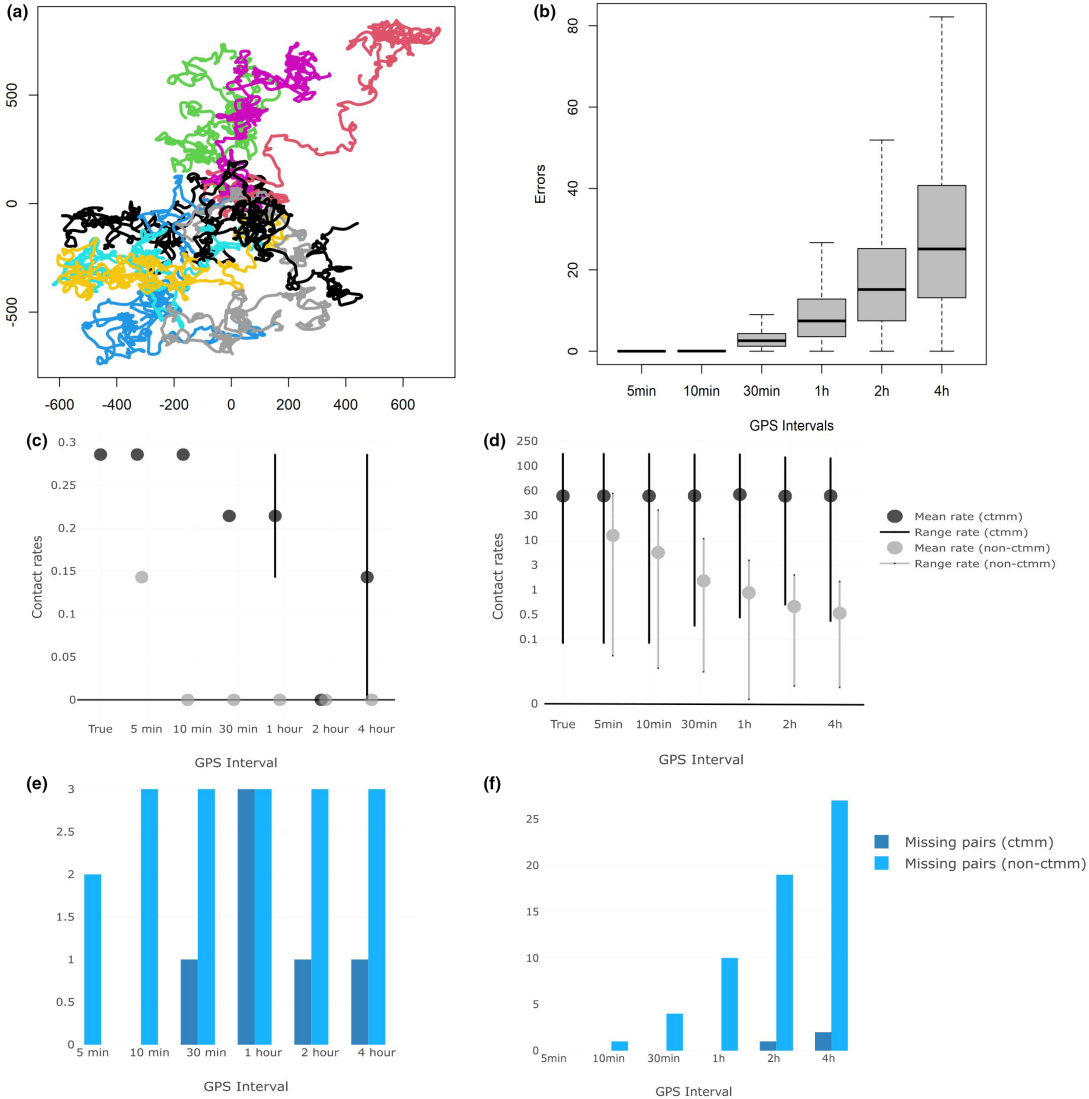
(a) Simulated, 1‐min interval trajectories for 10 animals. (b) Absolute differences in distances (spatial error) between true (simulated) trajectories and the predicted trajectories using the downsampled data with different temporal resolutions. (c and d) Mean and confident interval for direct (indirect) interaction rates using downsampled and CTMM interpolated data. (e and f) Number of missing pairs using resampled and CTMM interpolated data.

We assessed whether our CTMM‐Interaction method could recover true interactions using simulations. We employed a functional movement model (Hooten & Johnson, 2017) to simulate 1‐min movement trajectories for 10 animals for a week (i.e., 10,080 locations per animal). The trajectories were simulated using a Gaussian kernel with shape parameter *φ* = 0.001, approximating Brownian motion (see Hooten & Johnson, 2017). Simulated trajectories were treated as the true, observed locations without locational errors (Figure 2a). We then downsampled the simulated 1‐min observations to temporal resolutions of 5, 10, 30 min, 1, 2, and 4 h. We estimated the direct and indirect interactions using both the true, observed trajectories and the downsampled trajectories. We defined direct interactions as two animals in the same location at the same time within a 2‐m buffer (i.e., the approximate average body length of an individual) and 1‐min time frame to account for different body positions. Indirect interactions were defined as two animals visiting the same location within a 2‐m buffer and a 5‐day interval (representing the epidemiologically relevant interaction in the case of ASF). To test the sensitivity of spatial buffers on the estimation of interaction rates, we varied the buffer from 2–5 and 10 m using the simulated data (Figure S1).

Next, we fitted the CTCRW models to all six of the downsampled trajectories for each simulated individual and predicted their movements every minute. Note that while the CTCRW model is a special case of functional movement models (Hooten & Johnson, 2017), the functional movement model we used to simulate the data was not a CTCRW. We did this to account for the very realistic possibility that empirical data we collect will not exactly follow a CTCRW, which should allow a more realistic evaluation of CTCRW model performance. We evaluated the performance of CTCRW models in each downsampled scenario by comparing the differences in distances between the predicted locations and true, observed locations. Higher errors indicated that our fitted CTCRW was doing a worse job predicting the true movement trajectory, with implications for correctly inferring interactions. Finally, we followed the same procedure to extract direct and indirect interactions using the predicted 1‐min trajectories and quantified the discrepancies between interaction kernels extracted from 1‐min true observations and CTCRW predicted 1‐min trajectories to assess the performance of our CTMM‐Interaction method.

### 2.4 Application of CTMM‐Interaction method to empirical systems

To illustrate the parameterization and application of our method in empirical systems, we applied the CTMM‐Interaction method to two empirical systems, which involve species (i.e., wild pigs and mule deer) with different movement ecologies and the potential to carry different pathogens with different environmental persistence times. Wild pigs can be involved in ASF, where the pathogen African swine fever virus (ASFv) can remain viable in swine carcasses for several months in cold weather and in the environment for days to weeks depending on strain type and temperature (Davies et al., 2017). Mule deer can be involved in the transmission of CWD caused by a prion that can persist in the environment for years (Rivera et al., 2019). It is noteworthy that ASFv was not present in the study area, and we did not have disease surveillance data from CWD in our study area.

#### 2.4.1 GPS data

We captured and deployed GPS collars on 20 adult wild pigs (15 females; 5 males; University of Florida IACUC protocol 201808495) on a 42.3 km^2^ cattle ranch in south Florida in April–September 2017. Between December 2010 and December 2013, we captured and subsequently recaptured 47 adult female mule deer (Colorado Parks and Wildlife IACUC protocol 17‐2008 and 01‐2012) using helicopter net gunning at the Piceance Basin in northwest Colorado. The GPS collars were programmed to record fixes every 30 min for wild pigs in Florida and every 30 min for mule deer (location errors were estimated on average of 6–10 m in the study sites). Details about the study areas and systems are reported in Northrup et al. (2021); Yang, Boughton, et al. (2021); and Yang, Schlichting, et al. (2021).

#### 2.4.2 Extracting CTMM‐interaction networks

For both systems, we used CTCRW model to interpolate from observed discrete‐time GPS data with all locations with Position Dilution of Precision values <10 (Sands et al., 2006; data summary in Table S1) to continuous‐time movement with fixes predicted every minute. For parameterization in wild pigs, we calculated interaction weights ($w_{i,j}^{1\to 2}$) for each pair of wild pigs as follows. For both direct and indirect interaction, we defined the spatial encounter function, $f_{d}$(.), as a piecewise function with a distance threshold of 10 m (the buffer distance of 5 and 15 m were also tested; see the sensitivity analysis in Figure S1). This threshold reflects the direct spatial co‐occurrence required for transmission of ASF (transmitted by ingestion of contaminated materials from infected pigs) by simply accommodating GPS error (~6–9 m) associated with the GPS locations. For direct interaction where ∆τ = 0, we defined the temporal encounter function, $f_{\delta }$(.), as a constant of 1; for indirect interaction, we define $f_{\delta }$(.), as an exponential decay function within a 5‐day interval, given the high virulent strain ASFv like Georgia 2007/1 isolate can only persist in the environment for ~5 days in warm climates (Davies et al., 2017).

$f_{d}\left(s_{2}\left(\tau_{j}\right),s_{1}\left(\tau_{i}\right)\right)=\left\{\begin{array}{c} 1;d_{1,2}\leq 10m\\ 0;d_{1,2}>10m \end{array}\right.$, $f_{\delta }\left(∆\tau \right)=\left\{\begin{array}{c} 1;∆\tau =0\\ e^{-∆\tau };0<∆\tau \leq 5\text{days}\\ 0;∆\tau >5\text{days}. \end{array}\right.$

In the CWD‐mule deer system, for each pair of deer, we calculated interaction weights ($w_{i,j}^{1\to 2}$) based on the same spatial encounter function ($f_{d}\left(.\right)$ also with a distance threshold of 10 m) given a similar transmission pathway and temporal encounter function $f_{\delta }\left(.\right)$ as a constant of 1 (no decay) given the long period of CWD prion survival in the environment in most conditions (Rivera et al., 2019). Our objective was to simply describe interactions (and not transmission) in both systems, so we did not include explicit pathogen acquisition or shedding rates.

$f_{d}\left(s_{2}\left(\tau_{j}\right),s_{1}\left(\tau_{i}\right)\right)=\left\{\begin{array}{c} 1;d_{1,2}\leq 10m\\ 0;d_{1,2}>10m \end{array}\right.$, $f_{\delta }\left(∆\tau \right)=1$

To define interactions using movement data at different temporal resolutions, we defined a direct interaction as a co‐location at the same time (∆τ=0) and an indirect interaction as a co‐location with at least a 1‐min lag. We then generated interaction networks for both empirical datasets with nodes as individual animals and weighted edges as the daily average of instantaneous “weight” of interactions. We developed a new approach to calculate the daily average of instantaneous “weight” of interactions because some traditional association indices or weights (Cairns & Schwager, 1987) do not perform well when indirect interactions occur when animals are not tracked simultaneously (Section 4: Appendix S1). The approach treats the daily frequency of indirect interactions as the accumulation of lagged co‐location interactions, whereby interactions were calculated on temporally lagged movement trajectories of the two animals involved, with the accumulation including as many lags as there are nonzero‐valued days in the temporal encounter function (Section 3: Appendix S1). We call networks built from inferred continuous‐time movement trajectories with interactions extracted at each minute “1‐min CTMM”‐interaction networks.

#### 2.4.3 Comparing CTMM and observed interaction network

As mentioned above, the traditional way of estimating interactions is to extract them from the observed GPS data based on predefined spatial and temporal encounter functions (Robitaille et al., 2018). To show the differences in the estimations of interactions that might be expected using two methods in real‐world systems, we compared the differences in interaction rates in both systems. It is noteworthy that this comparison is not the assessment of our method.

For both systems, we defined direct interactions as co‐locations of GPS fixes within a 10‐m buffer and 1‐min gap. However, fixes were not always recorded precisely at 30‐min because it takes GPS receivers varying amounts of time to receive the satellite signals depending on the microhabitat the collared animal is in, resulting in “collar drifting.” To address the collar drifting issue, we also tested the sensitivity of our networks to other temporal encounter functions and found mild changes in the estimation of interactions by varying temporal encounter functions (Figure S1). We defined epidemiologically relevant indirect interactions for both systems following the same parameterization as the CTMM‐interaction method but using observed GPS data. Similarly, we considered the potential pathogen decay as we did for the 1‐min CTMM‐interaction estimates above and construct the interaction network with edges weighted by the average daily interaction “weights” extracted from observed fixes. We refer to the empirical summary of interactions using only the observed fixes as the “observed” interaction network.

Interaction weights calculated for the observed interaction network were based only on location recordings gathered at 30‐min or hourly intervals. Our indirect interaction networks are based on the sum of all time steps within an appropriate lag, so the 1‐min CTMM‐interaction network could potentially be larger because it contained 30 (or 60) terms for each term in the observed network. To make the interaction weights comparable, we “downsampled” the 1‐min CTMM‐interaction events to match the time interval of the observed GPS data. We aggregated any interaction detected at the 1‐min scale within the first half of the interval (within the first 15 min of a 30 min interval, e.g., 13:12) to the previous time step (e.g., 13:00) and the events that occurred within the second half of the interval (e.g., 13:25) to the next time step (e.g., 13:30). Within each half‐hour interval, “downsampled” interactions were assigned a value of 0 if no interactions were detected at the 1‐min scale in the corresponding half‐hour, and a 1 if any interactions were detected. To avoid confounding due to differences in sampling intensities, we limited our comparison to the downsampled CTMM and the observed network (we did not compare the 1‐min CTMM and observed network directly). We refer to the downsampled version of the 1‐min CTMM‐interaction method where interactions were calculated using the same fix interval as the observed approach but on the inferred continuous‐time movement trajectories—30 min—as “downsampled CTMM” interactions. See definition of interactions based on three methods in Table 1. We also calculated several network metrics relating to disease transmission to show the differences in network structures using different methods to estimate interaction (see Section 4 and 5: Appendix S1)

**TABLE 1:** Concepts of different types of interactions based on three methods.

Methods	Direct interaction	Indirect interaction
1‐min CTMM	Co‐location of hosts within 10 m buffer at the same time extracted from CTMM interpolated trajectories	Co‐location of hosts within 10 m buffer at different times extracted from CTMM interpolated trajectories and weighted by possible pathogen decay
Observed	Co‐location of hosts within 10 m buffer at the same time extracted from observed GPS fixes	Co‐location of hosts within 10 m buffer at different times extracted from observed GPS fixes and weighted by possible pathogen decay
Downsampled CTMM	Downsampled CTMM direct interaction to match temporal resolution of observed fixes	Downsampled CTMM indirect interaction to match temporal resolution of observed fixes

## 3 RESULTS

### 3.1 Performance of CTMM‐interaction method in simulations

**FIGURE 3:**
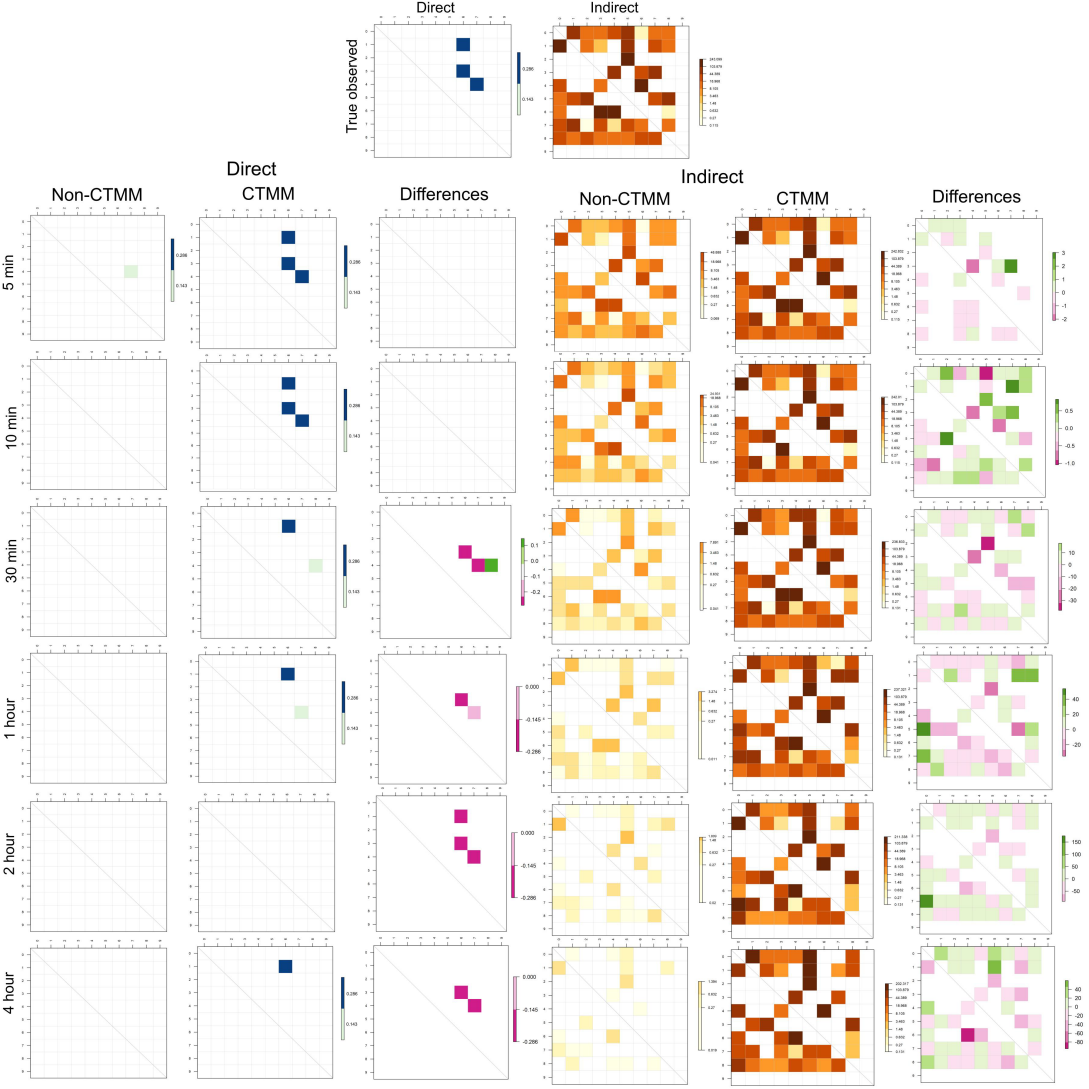
Direct and indirect interaction rates extracted from true, observed trajectories, the downsampled trajectories without CTCRW interpolation, and CTCRW predicted movements based on the downsampled trajectories in simulation cases.

We found substantial underestimations of direct and indirect interactions when the temporal resolution of the movement data becomes coarse compared with the true, observed data (Figure 2). When the temporal resolution was reduced to 5 minutes per fix, which in empirical cases is still in high resolution, the indirect interaction rates were underestimated ~5 times (Figure 3). No interactions were detected when the temporal resolution was every 4 h.

We also found that the performance of CTCRW varied by the resolution of the input movement data. The predictions were relatively accurate when movement data were recorded every 5 or 10 min. Considerable prediction errors in the movement trajectory (Figure 2) appeared when the fix rate resolution was greater than 30 minutes. Furthermore, those prediction errors also impacted the estimation of interaction rates. Errors in interaction estimates can be underestimates if the two interacting animals were predicted to be further apart (false negative), or overestimates if the two noninteracting animals were predicted to be close to each other (false positive; Figure 3).

### 3.2 Transmission matrix and spatial distributions

**FIGURE 4:**
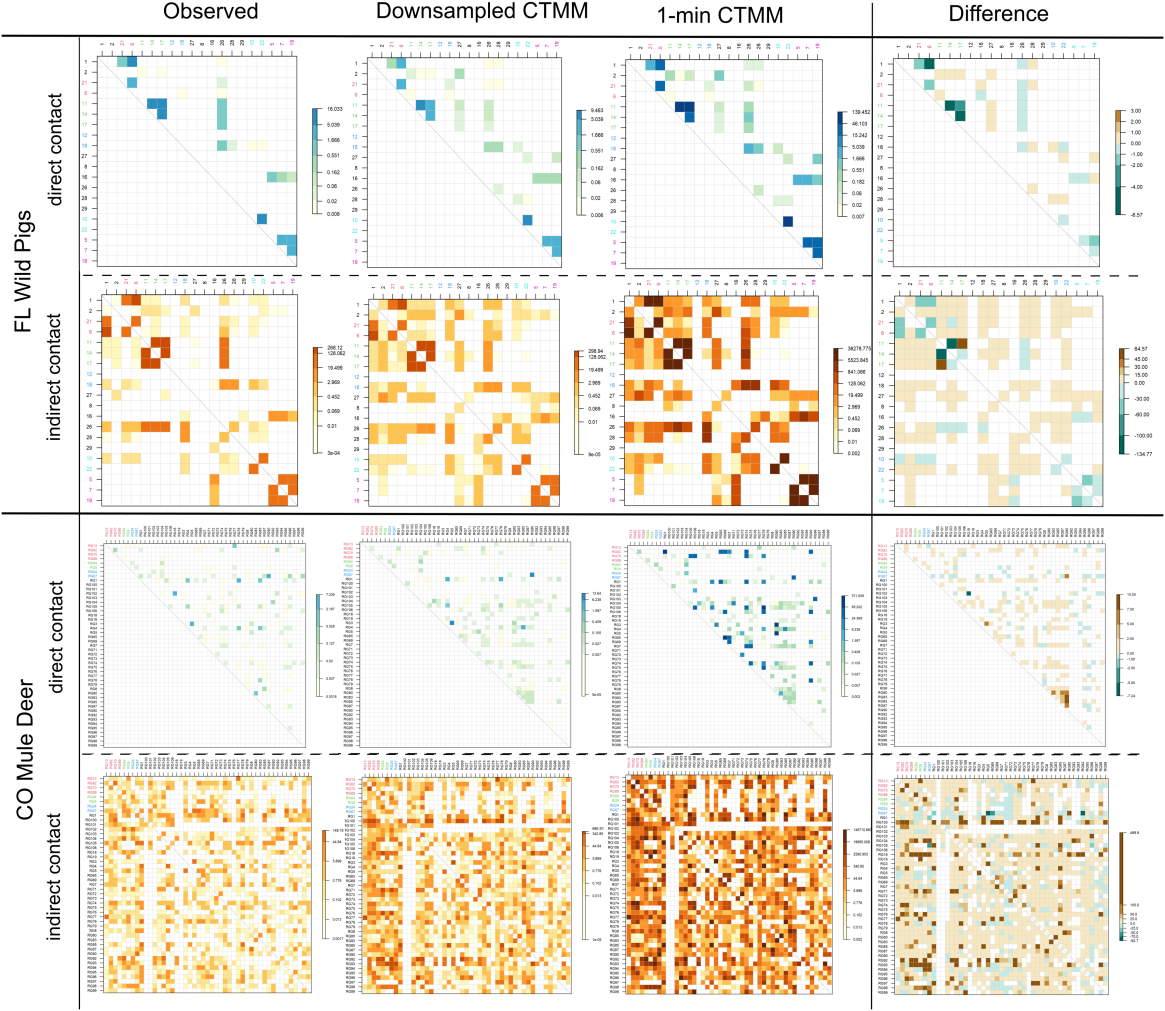
Pairwise weighted interaction matrix in two empirical systems. “Observed” and “1‐min CTMM” columns rely on transmission kernels estimated from interactions extracted from the observed GPS data with 30‐min interval and the interpolated continuous trajectory with 1‐min interval from CTCRW model, respectively. The “downsampled CTMM” column relies on a transmission kernel calculated using interactions that are downsampled from a 1‐min event to a 30‐min event to compare with the temporal resolution of interactions extracted from the “Observed.” The “Difference” column is the differences between transmission kernels of the “downsampled CTMM” and “Observed” columns. A positive number in the “Difference” column indicates that the downsampled CTMM‐interaction weight is higher than the observed interaction weight, while a negative value indicates that the downsampled CTMM‐interaction weight is lower than the observed interaction weight. The individuals tagged in the same colors indicate that they come from the same social groups.

In empirical systems, higher interaction rates and more interacting pairs were identified in the downsampled CTMM than in the observed interaction network in both systems (Table 2; Figure 4). This was consistent with our simulation analysis. Differences between the downsampled CTMM and observed graphs were greatest for indirect interactions (i.e., 785 unique indirect interaction pairs in the downsampled CTMM vs. 626 in the observed graph for mule deer; 87 unique indirect pairs in the downsampled vs. 54 in the observed graph for wild pigs). However, we also found the downsampled CTMM‐interaction network underestimated some weighted direct interaction rates between pairs within the same social groups in wild pigs relative to the observed method (Table 2; Figure 4).

**TABLE 2:** Comparisons between downsampled CTMM and observed method in the number of unique animal pairs in two systems.

Systems	Direct contact^a^		Indirect contact^b^	
	Observed method missed	Downscaled CTMM method missed	Observed method missed	Downscaled CTMM method missed
FL pig	14	2	66	0
CO Deer	91	43	398	78

	Weights_downscaled_ > Weights_observed_ ^c^	Weights_observed_ > Weights_downscaled_ ^d^	Weights_downscaled_ > Weights_observed_	Weights_observed_ > Weights_downscaled_
FL pig	21	19	141	33
CO Deer	192	60	1385	262

^a^ For unique animal pair with direct interaction, Host A interacts Host B is the same as Host B interacts Host A. Thus, Pair_*A*→*B*_ = Pair_*B*→*A*_.

^b^ For unique animal pair with indirect interaction, Host A feels the interaction with Host B is a different scenario from Host B feels the interaction with Host A. Thus, Pair_*A*→*B*_ ≠ Pair_*B*→*A*_.

^c^ The number of unique interaction pairs where the daily average of interaction rates weighted by pathogen decay extracted from downsampled CTMM are larger than observed method.

^d^ The number of unique interaction pairs where the daily average of interaction rates weighted by pathogen decay extracted from the observed method are larger than downsampled CTMM.

**FIGURE 5:**
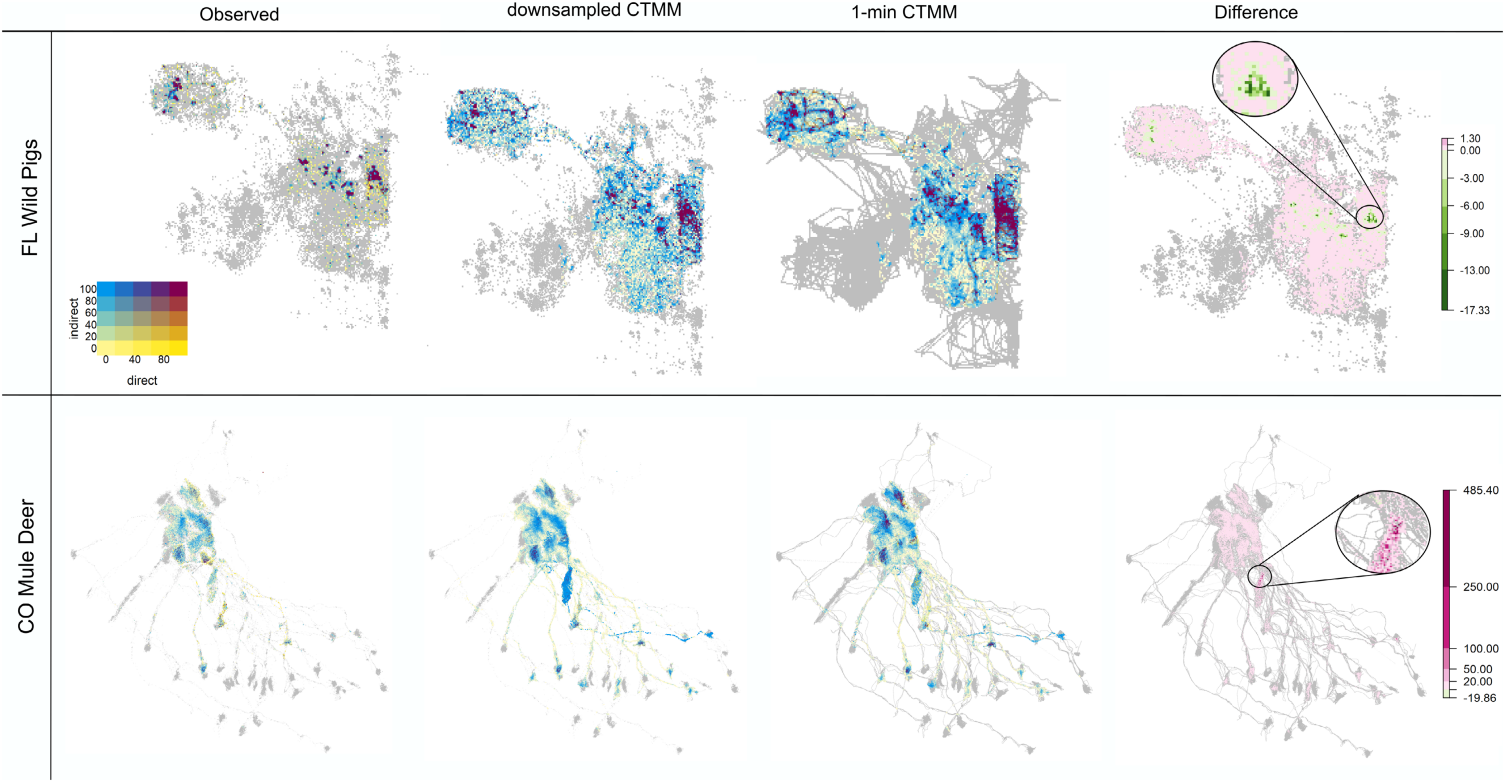
Spatial distribution of intensity of weighted direct and indirect interactions in two empirical systems. Labels along the top are as described in Figure 4. A positive number in the “Difference” map indicates that the intensity of the downsampled CTMM‐interaction weight at the location is higher than the observed interaction weight, while a negative value indicates that the intensity of downsampled CTMM‐interaction weight at the location is lower than the observed interaction weight. In the Difference column, we highlighted some areas with variations in differences between the downsampled CTMM‐interaction weights and observed interaction weights in magnified sections.

The spatial distribution of 1‐min and downsampled CTMM‐interactions covered three times the area of those from the observed method in both wild pig systems, although some underestimates of spatial interaction rates were found within social groups (Figure 5). In the CWD‐mule deer system, the 1‐min and downsampled CTMM‐Interaction methods identified 9% more interaction area compared with the observed method. The additional area where interaction occurred was primarily located along migration routes. Network metrics were calculated in Table 3, and more details about network structure can be found in Supporting Information.

**TABLE 3:** Network metrics summarized in two empirical systems with direct and indirect interaction weights combined.

FL wild pig	observed	downsampled CTMM	1 min CTMM
Modularity	0.22	0.20	0.21
transitivity	0.69	0.74	0.74
Edge density	0.28	0.46	0.46
Outdegree	NB (5.94,5.40), *x* = [0,10]	NB (*x*, mean = 11.58, var = 24.95), *x* = [0,14]	NB (*x*, mean = 11.58, var = 24.95), *x* = [0,14]
Indegree	NB (4.23,5.40), *x* = [0,10]	NB (*x*, mean = 7.26, var = 13.90), *x* = [0,14]	NB (*x*, mean = 7.26, var = 13.90), *x* = [0,14]
Outstrength	Exp (*x*, *λ* = 0.006), *x* = [0,528.69]	Exp (*x*, *λ* = 0.012), *x* = 0, 251.62]	Exp (*x*, *λ* = 5.86 e−5), *x* = [0, 58735.53]
Instrength	Exp (*x*, *λ* = 0.006), *x* = [0,543.01]	Exp (*x*, *λ* = 0.012), *x* = 0, 243.63]	Exp (*x*, *λ* = 5.86 e−5), *x* = [0, 59839.9]

CO Mule Deer			
Modularity	−0.004	−0.02	0.02
transitivity	0.78	0.88	0.88
Edge density	0.59	0.73	0.73
Outdegree	NB (*x*, mean = 26.91 var = 42.31), *x* = [10,38]	NB (*x*, mean = 33.38, var = 56.84), *x* = [15,43]	NB (*x*, mean = 33.38, var = 56.84), *x* = [15,43]
Indegree	NB (*x*, mean = 26.91 var = 42.31), *x* = [2,42]	NB (*x*, mean = 33.38, var = 57.85), *x* = [2,46]	NB (*x*, mean = 33.38, var = 57.85), *x* = [2,46]
Outstrength	Exp (*x*, *λ* = 0.009), *x* = [1.95, 496.03]	Exp (*x*, *λ* = 0.001), *x* = [0.89, 2954.89]	Exp (*x*, *λ* = 1.30 e−5), *x* = [71.98, 308836.80]
Instrength	Exp (*x*, *λ* = 0.009), *x* = [0.04, 559.50]	Exp (*x*, *λ* = 0.001), *x* = [27.22, 6149.5]	Exp (*x*, *λ* = 1.30 e−5), *x* = [2478.73, 239829.48]

## 4 DISCUSSION

Intra and interspecific interactions are keystone elements impacting various ecological processes, but how to quantify absolute interaction rates in space and time remains a challenge (Noonan et al., 2021). This study developed a novel method for deriving interaction networks from GPS telemetry data. Through simulation analysis, we found that our CTMM‐Interaction method could recover underlying interactions that were not directly observed. Moreover, in both simulation and empirical cases, the observed method underestimated interaction rates and spatial distributions and revealed different network topologies relative to the CTMM‐interaction method. The 1‐min CTMM‐interaction network also revealed substantially more interaction events, particularly low‐weight indirect interactions, leading to more integrated interaction networks with higher weighted interaction rates, distributed across larger spatial domains than interactions detected under the two coarser models.

### 4.1 Why leverage telemetry movement data?

In animal systems, interactions are usually quantified by animal monitoring technologies, such as camera traps, ultra‐high‐frequency proximity loggers, and GPS collars. Camera trap arrays monitor the interactions and behaviors of animals along with their visits to resources in the field of view (Kukielka et al., 2013), but generally provide data only within an observed area. Animal‐borne proximity loggers provide information on both the rates and duration of interaction events but lack spatial information (Boehm et al., 2009). Alternatively, passive integrated transponder tags placed at known resources of interest can estimate direct and indirect interaction rates surrounding that point resource but leave unresolved uncertainty in interaction rates at other locations. For small mammals, individuals sequentially trapped in the same location are assumed to have more interactions. However, sparse trapping data often preclude robust interaction estimation as interaction information is strictly limited to trapping sites and not across an animal's movement trajectory. GPS telemetry technology allows the monitoring and tracking of animal movement and spatially explicit interaction between collared individuals for long periods of time, but descriptive estimates from the raw data are limited temporally by the collars' fix rates. The observed method which only summarizes the interactions using the GPS fixes inevitably results in missed interaction events occurring in the gaps between GPS fixes. Additionally, it is common that multiyear or multisite animal tracking studies incorporate GPS data collected at different spatiotemporal resolutions (e.g., Yang et al., 2019), which can result in different definitions, precision, and accuracies of interaction estimates (Gilbertson et al., 2021). CTMMs allow prediction of animal movements between fixes, which helps to standardize the temporal resolution of the data across study periods and sites.

GPS‐derived interaction networks have been widely used in animal disease systems to estimate interaction rates across the landscape and infer disease transmission dynamics. However, overcoming the limitations of sampling on interaction estimates and determining transmission‐relevant interactions remain challenges that our approach provides an important step toward alleviating. Fully leveraging animal movement data to identify direct and indirect interactions could help pinpoint potential low‐use, but high‐influence areas (e.g., low interaction rate areas on habitat corridors) on a landscape that drives pathogen spread (Kauffman et al., 2022). Such areas can be important in disease systems where hosts get infected even with a small dose of pathogen (e.g., anthrax, Turner et al., 2014; Yang, Proffitt, et al., 2021).

Our CTMM‐Interaction framework can also be used to address questions in other ecological systems. For instance, understanding the dynamics of absolute interaction rates and durations between consumer and resources on the landscape might provide insights about functional responses to improve resource management (Lafferty et al., 2015). Estimating fine‐scale spatiotemporal interactions in group‐living species can also help identify the hierarchies in social organizations and underpinnings of collective behaviors or movements (Herbert‐Read et al., 2013). Additionally, our method helps to capture some instantaneous interactions, like predation‐ and transmission‐related interaction.

### 4.2 Why implementing CTMMs before the estimation of interactions?

Substantial underestimations of direct and indirect interactions were found in simulation cases when the temporal resolution of the movement data was coarse compared with the true, observed data. Our findings suggested that the performance of CTMMs depended on the resolution of observed GPS data with considerable prediction of locational errors found when the fixes were recorded greater than 30‐minute intervals. Despite potential locational errors introduced when fitting the “wrong” movement model to observed movement data, the predicted continuous trajectories can still recover true interactions, particularly if the resolution of the observed data is not too coarse (30 min fixes or less in this case). Importantly, fitting the CTCRW to 30‐minute observed data and inferring 1‐minute trajectories led to better approximations of the true interactions than trying to infer interactions from the 30‐minute data on its own. This has broad implications for our approach, indicating that even when we do not correctly specify the “true” movement model (which we will never do in practice) we can still recover true interaction rates.

Our simulated results highlighted the importance of incorporating high‐frequency movement data in interaction estimation. Although there could be some misidentification or underestimation in interactions due to the performance of CTMMs, our CTMM‐Interaction approach still captured the majority of interactions even with 4‐hour interval data in the simulations. Additionally, our results suggested that the interaction rates were sensitive to the selection of buffers, indicating the importance of selecting appropriate and biologically meaningful parameters (Figure S1). Also, it is noteworthy that the selection of a spatial buffer may also need to consider the locational errors of GPS fixes as we did for the empirical cases.

### 4.3 Potential errors and uncertainties

Several sources of error contribute uncertainty to interaction estimates from GPS data. First, error related to the model structure (e.g., independent identically distributed processes, Brownian motion, and Ornstein–Uhlenbeck process) and variability in CTMM prediction. We used a CTCRW model, which is a stochastic random walk model and therefore has error associated with each interpolated movement (Johnson et al., 2008). However, there are several approaches for fitting CTMMs, ranging from mean‐field continuous‐time stochastic processes (Calabrese et al., 2016) to mechanistic processes that depend on energetics (e.g., Hooten et al., 2019) or other underlying factors. These approaches have different sources of prediction uncertainties and performance. Selecting a CTMM appropriate to the system's biology (species, movement type, and environment) is important for generating reliable interactions (Martinez‐Garcia et al., 2020). Additionally, the interaction between movement ecology (e.g., average movement speed), temporal data resolution, and length of tracking period will influence the definition and estimation of interactions. The optimal interplay between these factors will be system specific, but at some point CTMM prediction error related to coarse, underlying data will dominate the uncertainty associated with inferred interactions. Our simulated scenarios also confirmed this with both false‐positive and false‐negative interactions detected (Figure S1). Additionally, we estimated the effects of CTMM prediction errors on our interaction inference by comparing interactions extracted from each fit from CTMM runs (i.e., either individual fits from different CTMMs or multiple predictions or parameterizations from one CTMM; see sensitivity analysis in Section 6: Appendix S1 as an example). We found that our results were robust to model structure and variability in CTMM prediction.

Second, interaction estimates can be sensitive to the parameterization of the interaction kernel and definitions of interactions (as discussed in the methods). We see this as a strength of our approach—the interaction kernel can be easily modified to capture known processes that affect ecologically and epidemiologically relevant interactions, for example, pathogen deposition rate decreasing with increasing host velocity. In our simulation assessments, overestimation increased in conjunction with enlarging the spatial buffer defining interactions (Figure S1). Therefore, we parameterized the spatial and temporal thresholds to extract direct and indirect interactions on both the biological process and the GPS location error within each empirical system showcased here. We conducted a sensitivity analysis on the spatial encounter function, $f_{d}\left(.\right)$, piecewise with distance thresholds of 5, 10, and 15 meters and found differences in the estimation of interaction kernels when using different thresholds, but our general conclusions were unchanged (Figure S1). When implementing our approach in other systems, researchers should explore how different definitions of interactions affect their inference on interaction structure and emergent ecological dynamics to ensure parameterization is appropriate.

Third, the locational error of GPS data can also shape uncertainty in the interaction estimates. When this is known to be a concern, new error‐informed CTMMs can account for GPS errors (Fleming et al., 2020). Additionally, GPS data combined with motion sensor‐derived information on heading and speed can be used to reconstruct fine‐scale movement paths (Gunner et al., 2021). Finally, the timescale of the CTMM interpolation can also impact the estimation of interactions. While the “optimal” timescale (e.g., 1‐min vs. 5‐min interpolation) will be a balance between the biology of the system and computational capacity, system‐specific knowledge can reasonably inform a biologically reasonable interpolation interval. Missing an interaction that is less than 1 minute in length might be irrelevant for some types of interactions (e.g., a consumer–resource interaction) such that a 1‐min interpolation is sufficient.

### 4.4 Limitations and future directions

As with all methods that estimate interaction rates, some of the interactions might not be relevant to the ecological processes of interest depending on animal behaviors. Particular host behaviors or environmental conditions at the time of interaction could have asymmetric effects on those processes (Manlove et al., 2022). A key advantage of our method is that by framing our estimation of interaction in terms of spatial and temporal encounter functions, it can be readily extended to integrate how animal behaviors, movement speed, and landscape covariates affect direct or indirect interaction rates. For example, in predator–prey systems surrounding land cover might greatly reduce the extent of the spatial encounter function (hunting in a forest might yield a smaller spatial encounter function than hunting in a grassland) and behavior of prey can impact predation success (e.g., crypsis might make prey temporarily unavailable despite being with the spatial encounter radius of a predator). In disease systems, foraging/ruminating behaviors might relate to pathogen acquisition and can be estimated by incorporating camera data or path segmentation approaches, offering a next step for future studies (Wilber et al., 2022). If directional data were available in tandem with animal movement data, we could incorporate an additional term in our interaction kernel that weighted the instantaneous weight of an interaction based on the direction two animals were facing.

The current CTMM‐Interaction method is a data‐driven method for estimating interaction heterogeneities in nature. We applied the method to wildlife disease systems; however, our approach can be adapted to other contexts where interactions among individuals play a key role. Because of its mechanistic underpinnings in movement and interaction ecology, CTMM‐Interaction can be expanded to incorporate a mechanistic linkage between interaction structure and environmental conditions and host demography. These mechanistic underpinnings allow the method to make predictions of interaction heterogeneities in other ecological settings using data from a subset of conditions—a much‐needed advancement in multiple fields in animal ecology.

## AUTHOR CONTRIBUTIONS

**Anni Yang:** Conceptualization (equal); data curation (equal); formal analysis (equal); methodology (equal); validation (equal); visualization (equal); writing – original draft (equal); writing – review and editing (equal). **Mark Quentin Wilber:** Conceptualization (equal); data curation (equal); methodology (equal); validation (equal); writing – review and editing (equal). **Kezia Manlove:** Conceptualization (equal); investigation (equal); visualization (equal); writing – review and editing (equal). **Ryan S. Miller:** Conceptualization (equal); writing – review and editing (equal). **Raoul Boughton:** Data curation (equal); writing – review and editing (equal). **James Beasley:** Data curation (equal); writing – review and editing (equal). **Joseph M. Northrup:** Data curation (equal); writing – review and editing (equal). **Kurt Vercauteren:** Data curation (equal); writing – review and editing (equal). **George Wittemyer:** Conceptualization (equal); project administration (equal); supervision (equal); writing – review and editing (equal). **Kim Pepin:** Conceptualization (equal); data curation (equal); project administration (equal); supervision (equal); writing – review and editing (equal).

## CONFLICT OF INTEREST STATEMENT

All authors declare no conflict of interest.

## Supporting information


Appendix S1.

## Data Availability

Data will be available on Dryad Digital Repository. Example codes are available at https://github.com/Anni‐Yang/ctmm‐interaction.
