# Supplementary material for: Deriving spatially explicit direct and indirect interaction networks from animal movement data
Source: Ecol Evol. 2023 Mar 26;13(3):e9774. doi: 10.1002/ece3.9774 (PMC10040956; doi:10.1002/ece3.9774)
Supplement: Supplementary file 1 — Appendix S1. [file ECE3-13-e9774-s001.docx]

***Supporting Information for***

**Deriving spatially explicit direct and indirect interaction networks from animal movement data**

Anni Yang, Mark Wilber, Kezia Manlove, Ryan Miller, Raoul Boughton, James Beasley, Joseph Northrup, Kurt C. VerCauteren, George Wittemyer, Kim Pepin

Fig. S1. Direct and indirect interaction rates extracted from true, observed trajectories using different spatial buffers.

Fig. S2. Pairwise weighted interaction matrix in wild pigs in FL using spatial encounter function as a piecewise function with a distance threshold of 5, 10, and 15 meters (See the PDF version below).

Fig. S3. Pairwise weight interaction matrix in two empirical systems based on different temporal gaps (i.e., 0 min, 5 min, 10 min, and 14 mins [maximum drifting gap]) to consider collar drifting problems. The interactions were extracted using observed GPS data.

**1. A toy example of the calculation of interaction kernels**

Here, we provide a toy example to illustrate the calculation of interaction kernels and interaction rates for two individuals. To simplify the computation, we used the observed GPS data for two individuals and assume that their GPS fixes recorded in every 30 minutes (48 fixes per day). Additionally, we assume that two individuals stay together at a location (site A) for 5 days. If we compute the interaction kernel for this scenario, we will have:

Animal 2

Animal 1

$$\begin{matrix} & t1 & t2 \end{matrix} \begin{matrix} t3 & \ldots& t240 \end{matrix}$$

$$\begin{matrix} t1 & w1,1 & w1,2 \end{matrix} \begin{matrix} w1,3 & \ldots& w1,240 \end{matrix}$$

$$\begin{matrix} t2 & 0 & w2,2 \end{matrix} \begin{matrix} w2,3 & \ldots& w2,240 \end{matrix}$$

$$\begin{matrix} t3 & 0 & 0 \end{matrix} \begin{matrix} w3,3 & \ldots& w3,240 \end{matrix}$$

$$\ldots\ldots$$

$$\begin{matrix} t240 & 0 & 0 \end{matrix} \begin{matrix} 0 & \ldots& w240,240 \end{matrix}$$

As we see from the kernel, *w* is the interaction weights, and the subscript is the time that animal 1 and 2 visited site A. Direct interaction will be the interaction events along the main diagonal of the kernel, while the indirect events are captured in the upper triangle of the matrix. If we assume all weights are equal to 1 (which for this example assumes that interaction weights do not decay with time), the indirect interaction weight for those 5 days will be equal to the sum of all weights except for those on the main diagonal, which will be 239*(1+239)/2=28,680 number of interaction events. A daily interaction rate will be 28680/5=5736 interactions per day.

**2 Weighted interactions in ecological processes**

Direct and indirect interactions can be defined as the co-locations of two individuals within a certain distance at the same time and at different times, respectively. The predefined “interaction” distance is often determined based on the mechanism of the ecological process of interest and the locational errors of the GPS data. For instance, disease transmission may require physical interactions, while collective behaviors (e.g., migration) may not. In some systems, the temporal gaps of indirect interaction (the time separating two individuals' occurrences at the same place) often impede description of the interaction process. For example, in predator-prey systems, the temporal gaps separating indirect interactions might determine predation success; in animal sociality, the gaps may affect group cohesion in collective movements; in infectious disease systems, the gaps can influence the transmission probability due to pathogen decay in the environment. Therefore, the instantaneous “weight” of an interaction is determined by spatial and temporal distances between two animals. Our methods will return all user-defined interactions for each pair of animals over the tracking period. However, post-hoc filtering may be needed for some cases. For example, in animal social networks, we might want to apply a minimum time together parameter to filter out random ‘fleeting’ interactions. Those filtering conditions and parameters can be defined by users before the integral process for summing interaction rates for each pair.

**3 Calculation of Overlap Period and daily interaction rates**

*New Method*

Traditionally, the edges of interaction networks are weighted based on the association index (Cairns & Schwager, 1987), daily interaction frequency and duration (Boehm et al., 2009), or spatial overlap proportions (Robert et al., 2012). However, there are some shortcomings for each of the conventional approaches, especially under indirect interaction situations. Association indices weight the total number of interactions by the number of samples or sampling periods that two individuals are both observed. Thus, they may not be directly applicable in the case where the two hosts were tracked at different time periods but still engaged in indirect interactions (Fig. S3). Spatial overlap between the individuals in Fig. S6 would not be captured by conventional association indices since the individuals’ trajectories never overlapped in time. In this situation, if only the observed tracking period were considered, there may be gaps between the tracking period of two hosts. Additionally, this situation allows use of daily interaction frequency to weigh the edges, since the daily interaction frequency is usually calculated as the number of interactions divided by the overlapped tracking period of hosts which is 0 this case.


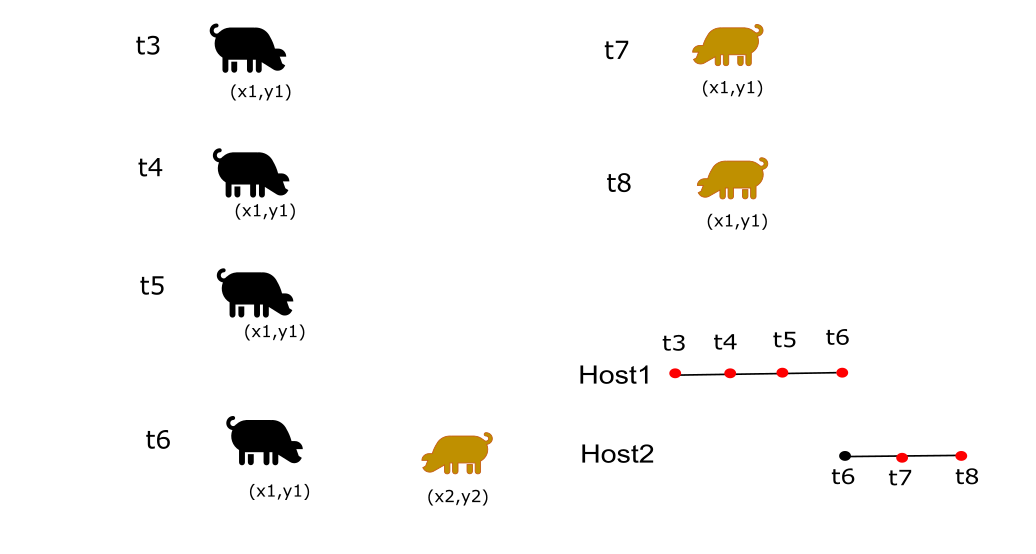


Fig. S3. Example in which the overlap period for potential interaction is not identical to the overlap in the animals’ tracking periods. Host 1 is shown as the black icon and host 2 is the gold. Each host’s coordinates at each timestep are listed below its icon. Both animals appear in t_6_, since t_6_ falls within both individuals’ tracking periods. In the two host trajectories, times when the two hosts overlapped in space (here, at location [x_1_,y_1_]) are shown with red points on each host’s timeseries in the lower right. Host 1 was at [x_1_,y_1_] for times t_3_-t_6_; host 2 was at that same location in t_7_ and t_8_, but not during timestep t_6_.

Here, we extend the traditional way of computing daily interaction frequency to adapt to the long-lagged indirect interaction situations that arise for pathogens that exhibit long environmental persistence (e.g., anthrax, brucellosis, and CWD) and also account for the pathogen decay rate. We calculate the edges of the interaction networks as the summed interactions weighted by pathogen decay and divided by the overlap period when potential interactions between two individuals could occur (i.e., the number of days). Particularly, we propose a new approach to account for time lag and the potential for asymmetric interactions in the calculation potential interaction overlap time. Assume that Host 2 experiences interaction with Host 1. For direct interactions, the numerator was the sum of all events in which individuals were located within the 10-meter distance of one another. The potential overlap time in the denominator was simply the period of time for which both animals were simultaneously tracked. The unit of the denominator can be adjusted to capture the rate of interest (i.e., daily, hourly, etc.). In the cases presented here, the rates were daily.

For indirect interactions, we calculated the “direct” interaction weight using that same approach on trajectories in which individual locations were examined at a series of progressively larger lags. For example, we lagged the trajectory of Host 2 one time-step (i.e., 1 day) back in each iteration until the lagged end of the tracking period of Host 2 was equal to the start of the tracking period for Host 1. At each lag, we calculated the summed number of (lagged direct) interactions as the numerator. The denominator at each lag scenario was the overlap time between the lagged trajectories of the two individuals, which is one day in the example below. To consider the pathogen decay in the indirect interaction rate, we then weighted the rate by an exponential decay function, $e^{-{\Delta t}_{lag}}$. Note that we chose an exponential decay function here for simplicity, but other pathogen decay functions could be used. Also, if data on behavior at the time of interaction are available, a second function could be added here to further weight the interaction by transmission risk. The total indirect interaction weight between two hosts was taken to be the sum of those lagged interaction weights over all time lags.

To explicitly illustrate the process, we provide a worked example as follows. Assume that on day 7 Host 2 is located at the same location where Host 1 was located on days 3 through 6 (Fig. S4). The total indirect interaction rate for Host 2 on day 7 and day 8 in location would take into account all the previous days that Host 1 located to the same point. For example:


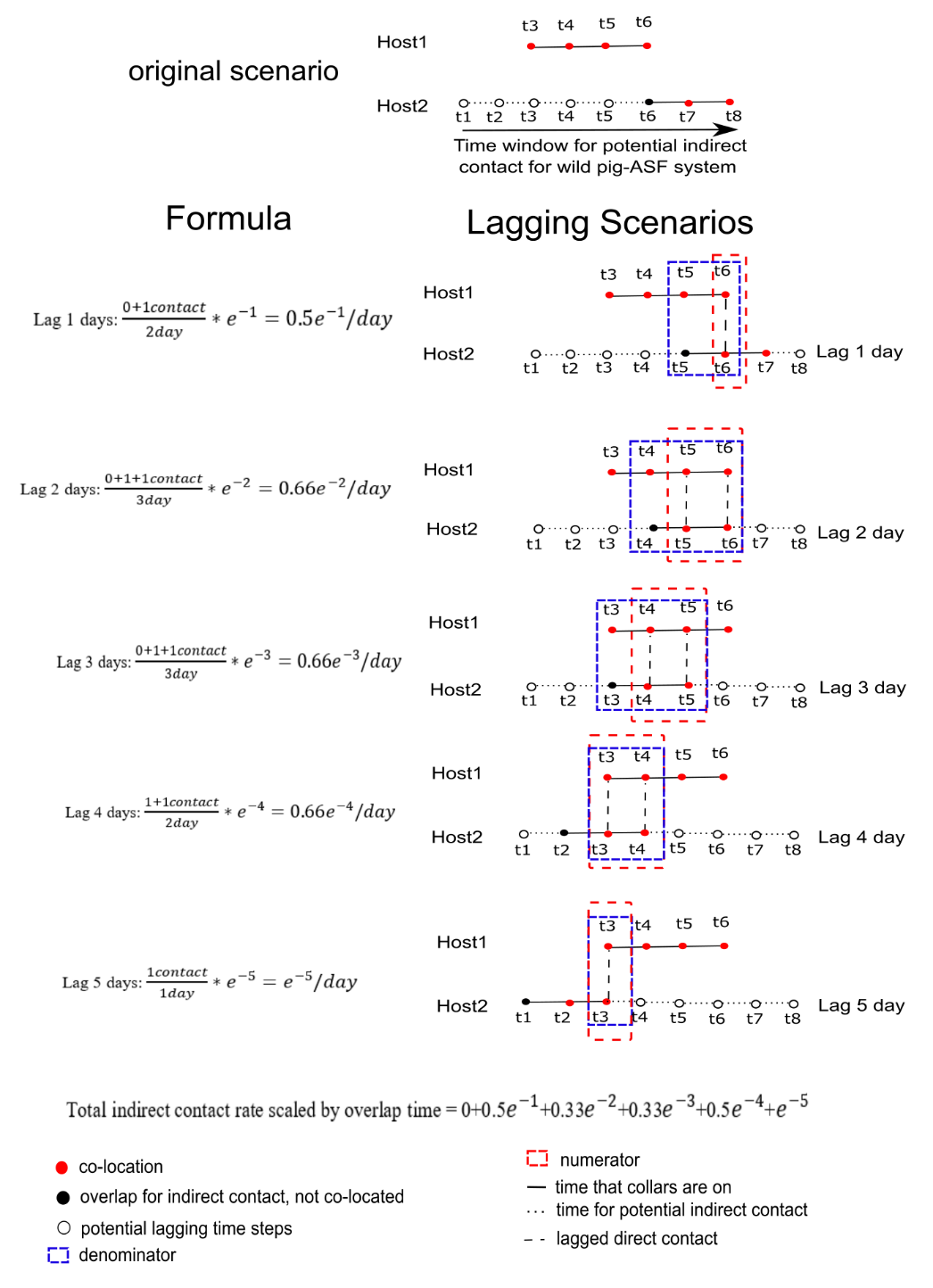


Fig. S4. The calculation of interaction rate scaled by the new overlap time and pathogen decay.

In general, if Host 1 was tracked between $t_{1s}$ and $t_{1e}$ and Host 2 was tracked between $t_{2s}$ and $t_{2e}$, assume $t_{1s}<t_{2s}<t_{1e} or t_{2e}$, the daily direct interaction rate will be calculated as:

$$W_{d}=No. direct contact/({t_{2s}-t}_{1e})$$

The daily indirect rate that Host 2 experiences interaction from Host 1 will be calculated as

$$W_{id}=\sum_{t_{lag}=0}^{t_{2e}-t_{1s}} \frac{No. lagged direct contact}{min\left( t_{1e},t_{2e} \right)-t_{1s}}*e^{-\Delta t_{lag}}$$

We used this approach to compute the interaction kernels for both the observed and CTMM- interaction to get the weighted interaction rates. The total computational time to estimate interaction kernels for SRS pigs, ABIR pigs, and CO deer were ~29h, 42h, and 159h on a 2-core, 64GB RAM computer.

*Potential caveat*

It is possible that our weighting scheme could overinflate interactions when there is little collar overlap time between pairs. For example, if both Host 1 and Host 2 visit the same location frequently but overlap time is low, we could be dividing a high numerator by a high denominator. To examine this potential false inflation of interaction rates that might be introduced by short overlap time, we made scatterplots of the patterns between overlap time and indirect interaction rates for each pair of hosts within two systems. We did not detect any patterns indicating that our method is relatively robust to this issue, at least for the datasets we examined (Fig. S5). There could be a positive correlation in ABIR sites but there were still many pairs having low indirect interaction rates with long overlap times of tracking.


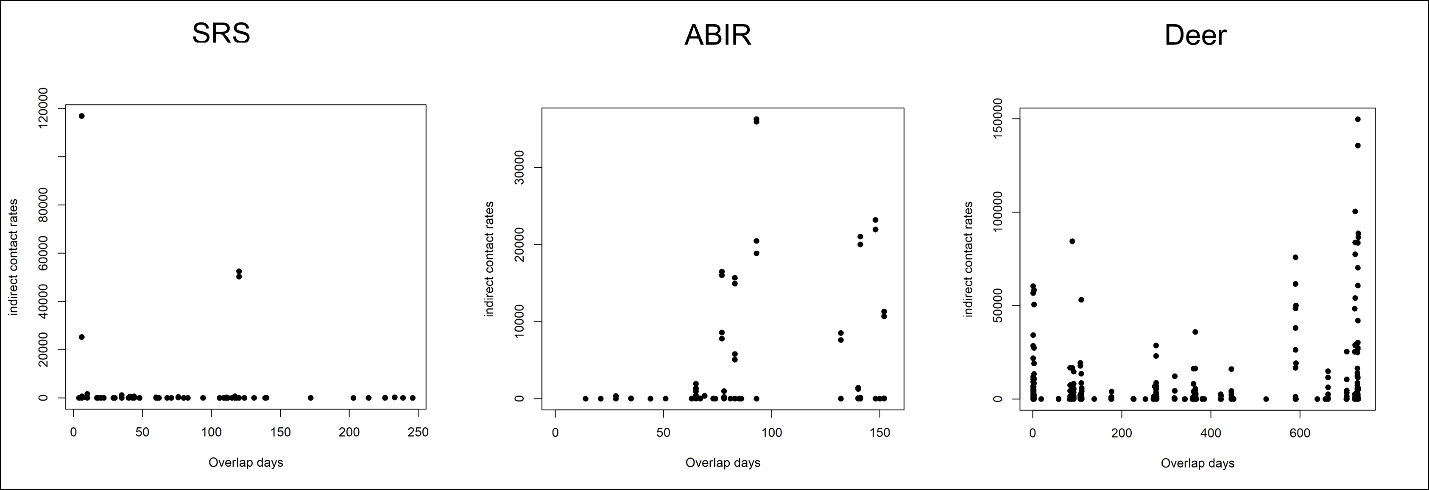


Fig. S5. The scatterplot of overlap times in days and indirect interaction rates for each pair of hosts for two study systems.

**4. Graph metric calculations**

We calculated several network metrics relating to disease transmission, graph topology, and group structure, including degree and strength centrality, modularity (strength of division of network into modules), transitivity (network connectivity), and edge density (ratio of the number of edges to the number of possible edges), for networks generated by all three methods (1-min CTMM, downscaled CTMM, and observed), and visualized their spatial distributions and network structures using the “igraph” package in R v4.1.1. Since both wild pigs and mule deer are group living species, we reported the interaction kernels and networks based on their social group memberships. Pairs with home range overlap greater than 50% are assumed to be in the same group (Yang, Schlichting, et al., 2021).

We found that more interaction pairs were identified under the 1-min CTMM-Interaction network, resulting in higher transitivity metrics and edge densities than those extracted from the observed interaction network. The distribution of indirect interaction strength was highly skewed in both scenarios, particularly in the CWD-mule deer system (Fig. S6; Table 3).


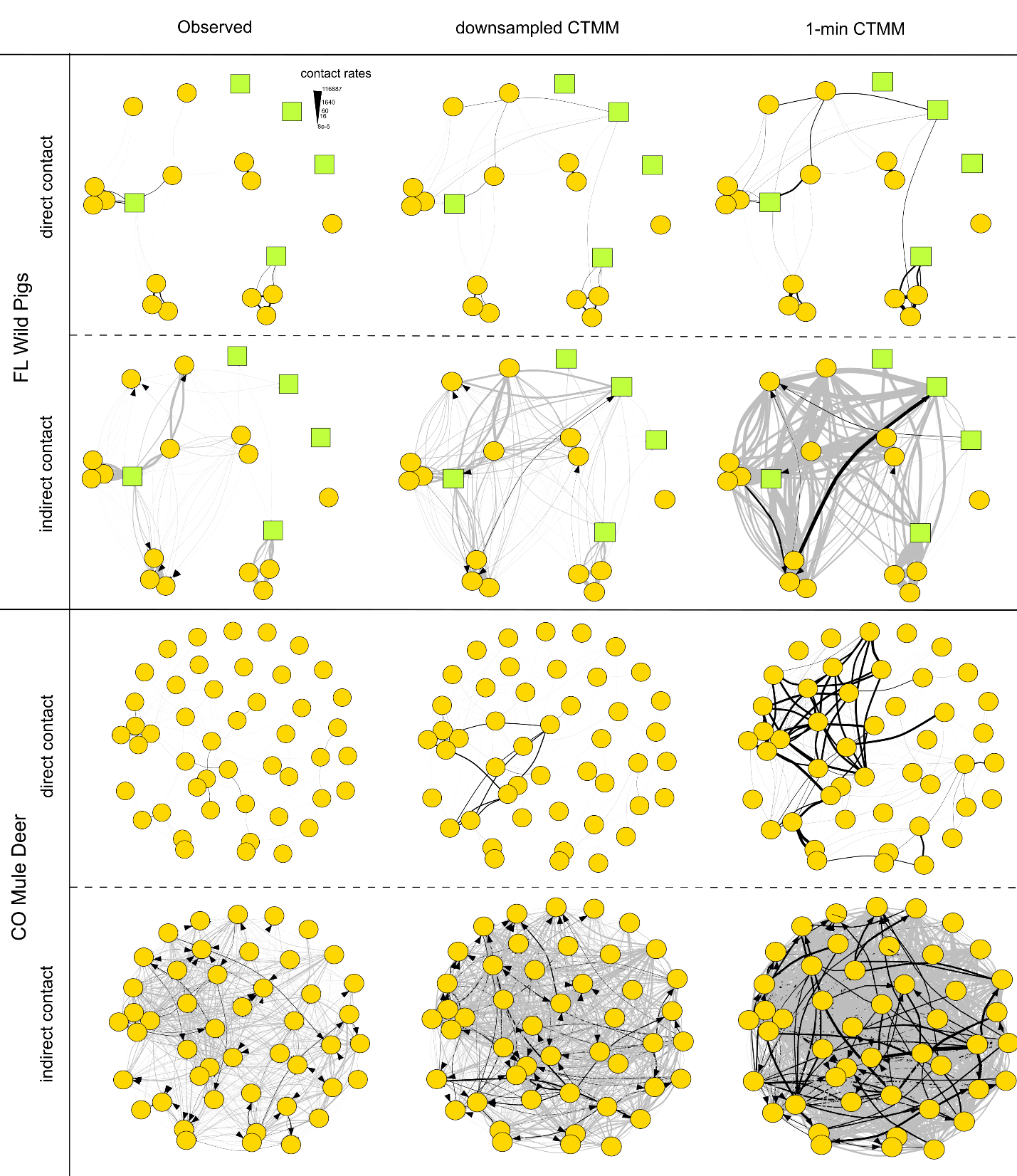


Fig. S6. Direct and indirect interaction networks in two empirical systems with edges weighted by transmission risk. Labels along the top are as described in Fig. 4. Yellow circles represent the female adults in the systems, while green squares are male adults. The gray edges in indirect interaction networks represent bi-directional interaction events (individual A interacts individual B, and individual B interacts A), while the black edges in indirect interaction networks represent directional interaction events (individual A interacts individual B, but individual B does not interact A). Individuals from the same social group were close to each other in the layout.

**5 System-specific biology**

Both CTMM and observed interaction networks suggested a strongly interconnected mule deer-CWD system in winter, indicating density-dependent CWD transmission in winter months (Storm et al., 2013). Such a pattern is likely due to a combination of long environmental persistence of CWD-causing prions (Dorak et al., 2017) and high levels of spatial overlap on deer winter home ranges (Northrup et al., 2021). A significant number of indirect interactions were identified along the migration routes in the CTMM interaction network, which could lead to frequency-dependent transmission in migration seasons. Such interaction patterns and transmission mechanisms were not detected by the observed method. Our method has the flexibility to adjust the definition of interaction to the temporal and spatial scale relevant to dynamics in the system (our definition of indirect interactions accounted for long persistence of CWD prions) and provides more refined and biologically consistent insight into interaction structure in a host-pathogen system.

In the ASF-wild pig example, both CTMM and observed interaction networks detected a high rate of within-group interactions in wild pigs and rare between-group direct interactions, which aligned with previous findings and can be explained by their social structure (Yang, Schlichting, et al., 2021). However, more between-group interaction was identified by the CTMM-interaction method than the observed method. Indirect interaction rates of wild pigs could be high, especially for males with large activity spaces that seek out interaction with multiple groups. With more between-group individuals connected in the CTMM-interaction method, the modularity of CTMM interaction networks were higher in both populations than the observed network. Interaction networks with high modularity could facilitate disease spread within groups. However, the spread of disease usually depends on between-group interactions. The structure of observed and absolute interactions can be different, which might lead to a different understanding of disease systems.

**6. Sensitivity analysis on the effects of CTCRW prediction uncertainties on interaction rates**

As mentioned in the Discussion section, CTMM prediction errors and uncertainties might impact the computation of interaction rates, and different CTMM models can have different prediction error structures. In this study, we employed CTCRW models, which include a continuous-time Ornstein-Uhlenbeck velocity process and a state-space framework to allow the random walk model to estimate parameters and predict animal locations during unobserved periods (Johnson et al., 2008). CTCRW also includes a particular error structure associated with each interpolated movement. Here, we present an example to explore how the potential CTCRW prediction uncertainties affected the identifications of interaction rates by using the subset of wild pig data from May 1 – May 14, 2017, at ABIR site. We followed the CTMM-interaction method to estimate direct and indirect interactions based on 100 predicted trajectories for each animal, from which we extracted the median interpolated trajectory used in the analysis in the main text. Maximum pairwise differences in direct and indirect interactions from the median were ~1% and ~2.6%, respectively (Fig S9). There was no significant variation in either direct or indirect interactions extracted from the CTMM interpolated trajectories considering prediction uncertainties given the Wilcoxon signed-rank tests (Table S1).


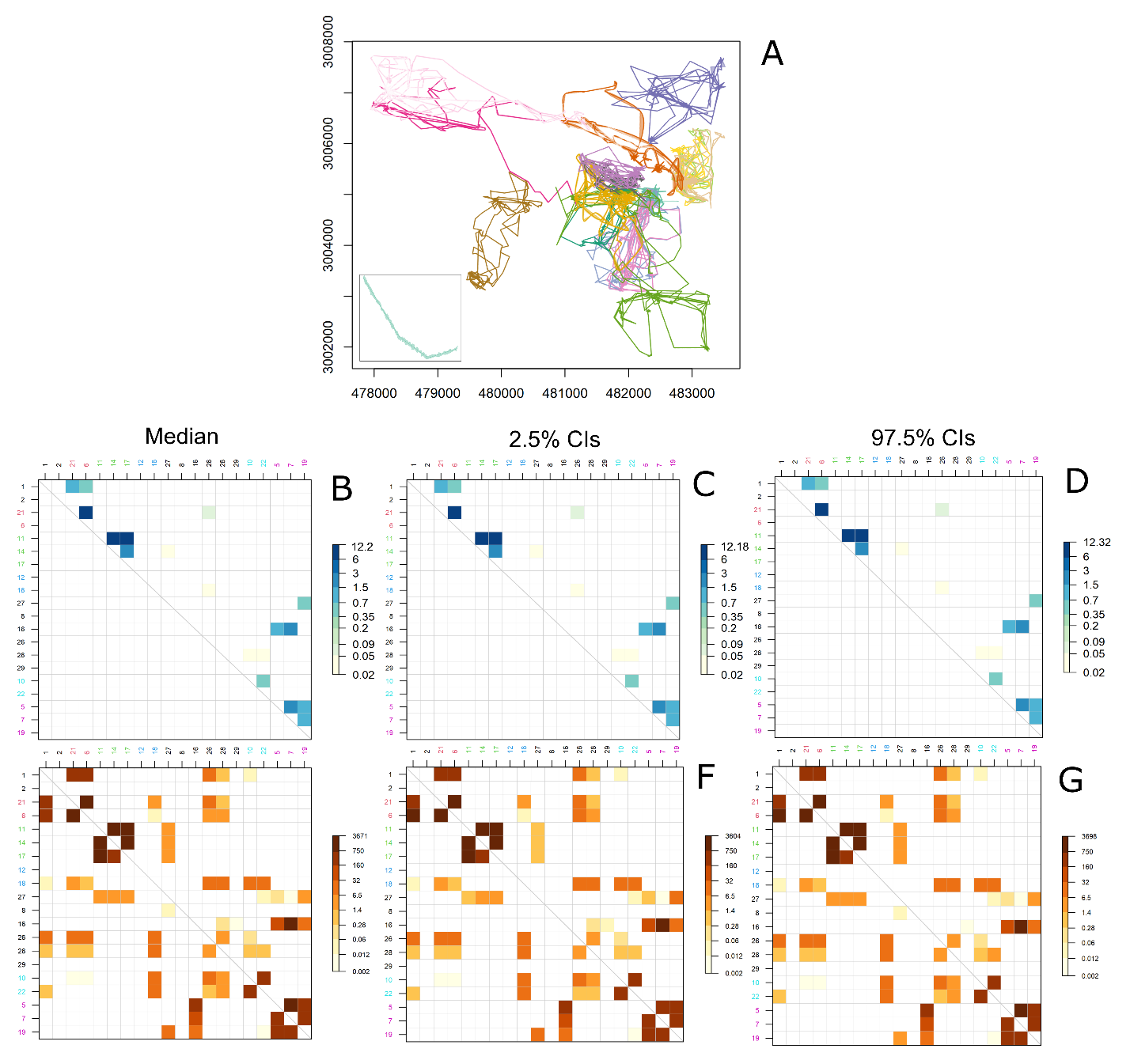


Fig. S7. Pairwise weighted interaction matrix extracted from wild pigs at ABIR site from May 1 – May 14, 2017. Panel A shows the median locations of the 1-min interpolated trajectories from 100 top selected CTCRW models and their 95% confidence intervals. The inset figure shows the zoom-in map for part of the trajectories for individual 8. Panel B is the direct interaction rates extracted using the median of the interpolated trajectories. Panel C and D are the 2.5 and 97.5 percentile of the direct interaction rates extracted based on all 100 CTCRW predictions. Panel E shows the indirect interaction rates extracted using the median of the interpolated trajectories. Panel F and G are the 2.5 and 97.5 percentile of the indirect interaction rates extracted based on all 100 CTCRW predictions.

Table S1. Summary of the number of the observed GPS fixes and the number of fixes left in the analyses for each case study

|  | Number of the observed GPS fixes | Number of fixes left in the analyses |
| --- | --- | --- |
| FL Wild pigs | 101088 | 100434 |
| CO Mule deer | 792037 | 770513 |

Table S2. Results of Wilcoxon test for comparing the 2.5% and 97.5% quantiles of the interaction rates extracted from all interpolated trajectories with the median values.

|  | Comparison | W | p-Value |
| --- | --- | --- | --- |
| Direct interaction | 2.5% VS median | 123 | 0.23 |
|  | 97.5% VS median | 162 | 0.99 |
| Indirect interaction | 2.5% VS median | 3174 | 0.85 |
|  | 97.5% VS median | 3805 | 0.99 |
